# Microarray analysis of genes and gene functions in disc degeneration

**DOI:** 10.3892/etm.2013.1421

**Published:** 2013-11-22

**Authors:** YANCHUN TANG, SHAOKUN WANG, YING LIU, XUYUN WANG

**Affiliations:** 1 Department of Rheumatism and Immunity, Yantai Yuhuangding Hospital, Yantai, Shandong 264000, P.R. China; 2 Department of Health, Yantai Yuhuangding Hospital, Yantai, Shandong 264000, P.R. China

**Keywords:** degenerative intervertebral discs, function enrichment, interaction network, drug-like small molecule, active binding site

## Abstract

The aim of the present study was to screen differentially expressed genes (DEGs) in human degenerative intervertebral discs (IVDs), and to perform functional analysis on these DEGs. The gene expression profile was downloaded from the Gene Expression Omnibus database (GSE34095)and included six human IVD samples: three degenerative and three non-degenerative. The DEGs between the normal and disease samples were identified using R packages. The online software WebGestalt was used to perform the functional analysis of the DEGs, followed by Osprey software to search for interactions between the DEGs. The Database for Annotation, Visualization and Integrated Discovery was utilized to annotate the DEGs in the interaction network and then the DEGs were uploaded to the Connectivity Map database to search for small molecules. In addition, the active binding sites for the hub genes in the network were obtained, based on the Universal Protein database. By comparing the gene expression profiles of the non-degenerative and degenerative IVDs, the DEGs between the samples were identified. The DEGs were significantly associated with transforming growth factor β and the extracellular matrix. Matrix metalloproteinase 2 (MMP2) was identified as the hub gene of the interaction network of DEGs. In addition, MMP2 was found to be upregulated in degenerative IVDs. The screened small molecules and the active binding sites of MMP2 may facilitate the development of methods to inhibit overexpression of MMP2.

## Introduction

Disc degenerative disease (DDD) is a common and frequently occurring orthopedic disease. The main clinical manifestations are disc herniation, vertebral instability and spinal stenosis. The intervertebral disc (IVD) is an immune-free organ that is completely closed, without vessels and nerves. Its immune privilege provides a potential environment for treatment with gene therapy. However, the etiology and pathophysiological mechanisms of DDD require further investigation (1).

Gene therapy is a novel therapeutic strategy that is capable of consistently changing and affecting the physiological function of cells. It has received considerable attention in the field of biological therapy, and promising developments have been made in this area (2). With regard to gene therapy for DDDs, depending on use of the appropriate dose and on the treatment area in the body, gene therapy is capable of producing positive therapeutic effects. However, devastating side effects are likely to arise if the therapeutic gene is not correct, if leakage occurs when the therapeutic genes are injected or if the dose is inappropriate. Therefore, an important aspect of gene therapy is determining the correct target gene (3).

Genes work in synergy with other genes to perform biological functions. They simultaneously interact with multiple genes and trigger a variety of changes that lead to diverse reactions. The functional annotation of regulated genes, using Gene Ontology, has enabled the identification of severely affected groups of genes that correlate with the disease phenotypes (4). Genes involved in mutation of the IVD are enriched in the Gene Ontology terms ‘multicellular organism development’ and ‘pattern specification’ (5).

A phenotype is the result of a series of complex molecular reactions. A protein interaction network embodies the complex interactions between genes (6–7). An abundant and organized network of elastic fibers has been previously observed in the non-degenerated human disc using immunohistochemical staining (8). In addition, Yu *et al*(9) revealed that an abundant and organized network of elastic fibers was present in adolescent (12- and 17-year-olds) human IVDs, and suggested that the elastic fiber network had a significant biomechanical function (9). Trp2 and Trp3 allelic products have been incorporated into the cross-linked fibrillar network to study the process of developing human cartilage (10).

Once the disease-related genes have been identified, the active binding site may provide another perspective for the treatment of the DDD. It has been demonstrated that the deletion of transforming growth factor β receptor 2 (Tgfβr2) in Col2a-expressing mouse tissue results in alterations in the development of the IVD annulus fibrosus (5). Based on the genes associated with the disease phenotype, drugs to repress these genes are screened. The Connectivity Map (CMAP) database is a collection of genome-wide transcriptional expression data that have been obtained from cultured human cells treated with bioactive small molecules and simple pattern-matching algorithms. These data enable the discovery of functional connections between drugs, genes and diseases through the transitory feature of common changes in gene expression (11). Yeh *et al (*12) screened drugs that targeted cancer stem cells (CSCs) to improve current treatment and overcome drug resistance. Gene signatures between embryonic and CSCs were used to identify potential drugs that were capable of reversing the gene expression profile of CSCs, based on CMAP.

The present study identified differentially expressed genes (DEGs) by comparing the gene expression profiles of non-degenerative and degenerative discs. The Gene Ontology terms enriched among the DEGs were screened. Following the construction of the interaction network of the DEGs, hub genes were considered as targets for gene therapy, and small molecules that were associated with DDD were studied to facilitate the treatment of DDD.

## Materials and methods

### Gene expression profiles of DDD

The gene expression profiles of DDD were assessed using the Affymetrix Human Genome U133A Array (HG-U133A) at platform GPL96 (Affymetrix, Santa Clara, CA, USA), which covered 22,283 probes in the human genome. The profiles were submitted by Zhou *et al* to the Gene Expression Omnibus (13). The samples included three degenerative and three non-degenerative IVDs from elderly patients and younger patients, respectively. The annotation information for the gene expression profile was downloaded, as well as the raw data.

### Data preprocessing and differential gene analysis

The original expression profile in CEL format was transformed into a matrix using affy package in R language (14–15). The median method was used for normalizing the expression matrix. Subsequently, the Limma package (16) was utilized to identify the differential genes between the degenerative and non-degenerative IVDs. The threshold for the P-value was set to 0.05 and |logFC| was set to 1.

### Enrichment analysis of differential genes

The WEB-based GEne SeT AnaLysis Toolkit (WebGestalt) was designed for the enrichment analysis of differential genes, and comprises a set of analytical tools for biological analysis. WebGestalt contains 59,278 functional categories derived from centrally and publicly curated databases, as well as computational analyses, including National Center for Biotechnology Information, Ensemble, Kyoto Encyclopedia of Genes and Genomes and Gene Ontology (17–18), for eight species, such as humans, rats and mice. In this study, the functional enrichment analysis was based on the hypergeometric test [false discovery rate (FDR), <0.05] for the DEGs.

### Constructing an interaction network of differential genes

Since one gene always acts in synergy with other partners, the interactive protein should also be studied when exploring the function of one gene and its protein (19). The Osprey platform (20) facilitates the study of protein-protein interaction networks and protein complexes by integrating the interaction information from the Biomolecular Interaction Network Database (21) and the General Repository for Interaction Datasets (22), which includes >50,000 of the interactions.

Therefore, Osprey software was used to analyze the interactions between differential genes and build the interaction network. Following topological analysis of the interaction network of differential genes, the genes with high degrees were screened as a hub. Hubs are to key maintenance of the integrity and robustness of the network and if specific hub genes are knocked out, the network is likely to be divided into fragments or even undergo paralysis (23).

### Functional enrichment analysis for differential genes in the interaction network

In gene enrichment analysis, a set of genes with similar or related functions are considered as a whole. By calculating the global and significant gene expression changes for the genes in the set, it is possible to determine whether the biological function has altered. This strategy greatly reduces the dimensions of the data; however, it makes the analysis much closer to the actual biological problems. Thus, gene enrichment analysis is popular in gene expression profiles (24). To date, there are numerous tools providing gene function enrichment analysis. In the present study, the Database for Annotation, Visualization and Integrated Discovery (DAVID) was used (25) for the functional enrichment analysis of the differential gene hubs in the interaction network. The FDR threshold was set to 0.05.

### Screening of drug-like small molecules

The DEGs in the interaction network were divided into two groups of upregulated and downregulated genes. By comparing the expression pattern similarities of the differential genes and genes perturbed by small molecules in the CMAP, small molecules involved in the disease were able to be identified (11,26). Small molecules with a score >0.8 were considered to be associated with the disease.

### Active site screening for hub genes

Proteins are the basis of life and are composed of ~20 amino acids. The structure of proteins is maintained by numerous interactions, including hydrogen bonds and ionic interaction forces. Due to the influence of these forces, protein molecules fold, forming a complex 3-D structure (27). In the molecular structure of the protein, the selective interaction region, which binds with other molecules, is referred to as the active site. The selective interaction between proteins is determined by the functional groups of the amino acids. The accurate prediction of a selective interactive region provides an enhanced understanding of specific protein interactions, which is of great significance for biologists (28). The Universal Protein (UniProt) (29) database stores comprehensive information regarding proteins, and is the most popular database for protein research. UniProt is integrated from Swiss-Prot, TrEMBL and the Protein Information Resource-Protein Sequence Database. In the current study, the active sites were screened for hub differential genes based on the UniProt database.

## Results

### Differential gene screening

**Figure 1:**
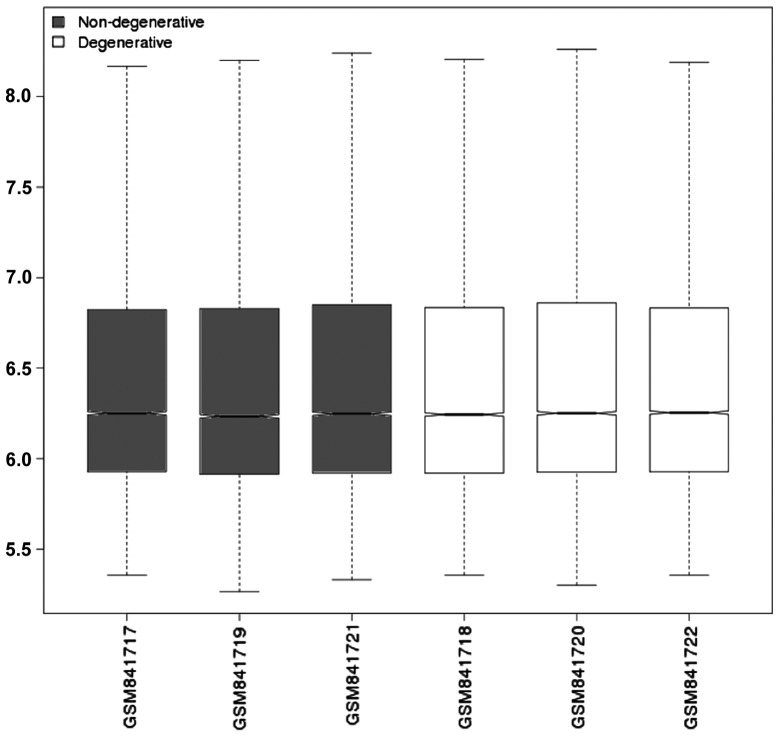
Median-normalized expression profiles. The grey and white boxes represent normal and degenerative intervertebral discs, respectively. The x-axis represents the sample and the y-axis represents the gene expression in the sample.

The gene expression profiles were normalized, as shown in Fig. 1. The median of each sample was the same, which showed that the data were well normalized (Fig. 1). When comparing the degenerative and non-degenerative IVDs, 53 DEGs were identified under the threshold of FDR <0.05 and |logFC| >1. A total of 16 genes were downregulated and 37 genes were upregulated. Matrix metalloproteinase 2 (MMP2) was upregulated in degenerative IVDs with its corresponding probe of 218468_s_at and the adjusted P-value of 0.04.

### Enriched terms among the DEGs

**Figure 2:**
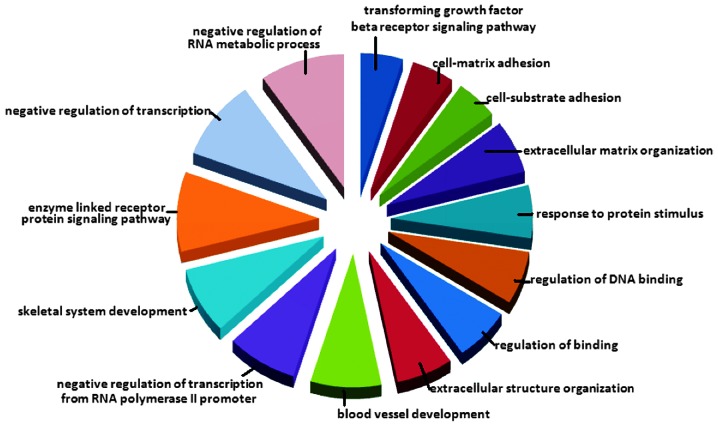
Enriched Gene Ontology terms for the differentially expressed genes.

The WebGestalt web tool was used for the enrichment analysis. As shown in Fig. 2, 14 Gene Ontology terms, including extracellular matrix, were enriched among the DEGs (Fig. 2).

### DEG interaction network

**Figure 3:**
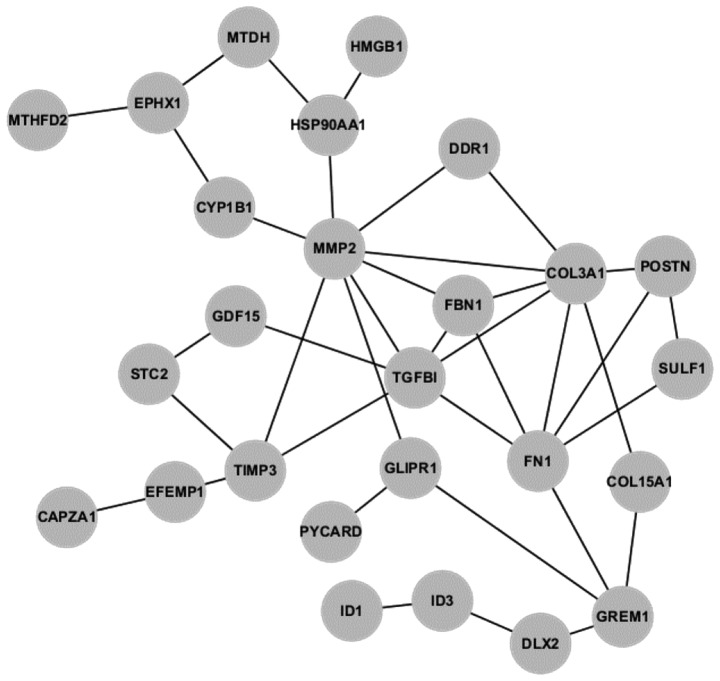
Interaction network of differentially expressed genes, constructed by Osprey software.

**Figure 4:**
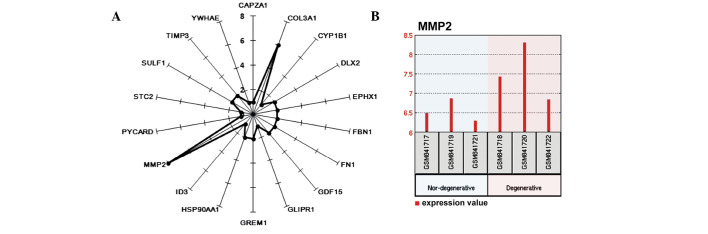
(A) Degree distribution for each gene in the interaction network. (B) The expression of the hub gene, MMP2, in all the six samples. MMP-2, matrix metalloproteinase 2.

Osprey software was utilized to explore the interactions among the DEGs. Fig. 3 shows the interaction network, which consists of 19 DEGs. Following construction of the network, the degree of each gene in the network was calculated. As shown in Fig. 4A, the gene MMP2 was connected to eight other genes, and was, therefore, the hub of the network (Fig. 4A). MMP2 was upregulated in degenerative IVDs (Fig. 4B).

### Gene Ontology terms enriched among the genes in the interaction network

In this study, DAVID was used to identify the Gene Ontology terms enriched among the genes in the interaction network. As shown in Table I, nine terms were significantly enriched. The most significant term was organ development, which was the only term associated with MMP2.

### Small molecules involved in degenerative IVDs

The DEGs were divided into two groups of upregulated and downregulated genes. Using the two gene groups as inputs for the CMAP database, the small molecules that were associated with the degenerative IVD were determined. The small molecules with a score of >0.8 were focused on for further analysis. As shown in Table II, the small molecule, MG-262, had the highest score (score=0.896).

### Hub gene active sites

For the hub protein in the interaction network, the UniProt database was searched for active binding sites of the MMP2 ligand. As shown in Table III, 22 metal ligand binding sites were revealed, with binding sites for metal ions, such as calcium and zinc.

## Discussion

In the present study, the gene expression microarray of non-degenerative and degenerative disc tissue samples was compared. The differential genes were screened out and their functions were further analyzed. The proposed method provides basis for the identification of candidate targets for the clinical treatment of DDD. It also facilitates clinical medication.

The results revealed that the DEGs were associated with TGF-β and the extracellular matrix, which was the fourth most enriched term. Degeneration of the IVD is predominantly a chronic process, involving the excessive destruction of the extracellular matrix (30). IVD degeneration has been demonstrated to be a progressive disease, mainly characterized by the alteration of the extracellular matrix composition (31). Factors involved in these processes have been suggested to be important for the identification of target genes in degenerative discs (32). In the interaction network of the DEGs, MMP2 was shown to have the highest degree. The MMPs are a class of proteolytic enzymes containing zinc and calcium, which are predominantly involved in the metabolism of the extracellular matrix. Roberts *et al*(33) suggested that MMPs were one of the primary factors leading to the disorder of the matrix structure and the degradation of the matrix in IVDs. The expression of MMPs has been shown to be significantly increased in the degenerative IVD, disrupting the balance between the MMPs and their endogenous inhibitor. In addition, it has been observed that MMPs are produced by IVD autologous cells and cells involved in invasive neovascularization in the IVDs. Therefore, Roberts *et al*(33) suggested that inhibiting the activity of MMPs may be an effective method of treating disc degeneration (33). Based on the downregulated and upregulated genes, six small molecules, which were most relevant to the degenerative IVD, were screened out. Hexabrix (ioxaglate), the most significant negatively-associated small molecule, has been introduced as a novel low-osmolality contrast agent for lumbar epidural double-catheter venography (34). Cortisone, which has been predicted to cure DDD, has been shown to result in satisfactory remission of articular facets syndrome, leaving the patient free from pain. Epidural cortisone injections are able to efficiently and safely release anterior and anteroposterior fusion for lumbar disc pain (34).

Further studies on activity sites may aid the inhibition and promotion of the normal expression of DEGs. Zinc metalloproteinases exert particularly important effects, directly and indirectly through the promotion of neovascularization. The zinc metalloproteinases closely interact with other metabolic factors to produce disc disorders (35). Calcium has been used as an indicator of calcification potential in human IVD degeneration and scoliosis (36).

At present, biological treatment must focus on the biological changes occurring in IVD degeneration, promote the synthesis of extracellular matrix and inhibit the reduction in levels of the extracellular matrix. In this manner, the metabolism of the extracellular matrix in the IVD is likely to return to normal. The mechanism underlying IVD degeneration is complex and has been suggested to correlate with changes at the molecular level. With an enhanced understanding of the disease from a molecular biology perspective, novel strategies are likely to emerge to prevent and treat degenerative IVDs. Although significant efforts have been committed to further elucidate the degenerative mechanism at the molecular and genetic levels, studies must be performed to evaluate the pathological roles of a number of factors and their interactions in the process of disc degeneration.

## Figures and Tables

**Table I:** DEGs in the interaction network.

GO-ID	P-value	x	Description
48513	0.004904	11	Organ development
48731	0.010540	12	System development
9653	0.015218	8	Anatomical structure morphogenesis
48856	0.015218	12	Anatomical structure development
7275	0.029394	12	Multicellular organismal development
32501	0.029394	15	Multicellular organismal process
48523	0.033435	9	Negative regulation of cellular process
9892	0.035170	6	Negative regulation of metabolic process
32502	0.040190	12	Developmental process

GO, Gene Ontology; DEG, differentially expressed gene.

**Table II:** Significant small molecules revealed by CMAP.

CMAP name	Score	P-value
Ioxaglic acid	−0.828	0.01022
Trazodone	−0.826	0.01052
Cortisone	−0.805	0.01462
Diperodon	0.817	0.01208
Procainamide	0.85	0.00074
MG-262	0.896	0.00218

CMAP, Connectivity map.

**Table III:** Search results for active binding sites.

Type	Binding site	Metal type
Metal binding	102	Zinc 2; in inhibited form
Metal binding	134	Calcium
Metal binding	168	Calcium 2
Metal binding	178	Zinc
Metal binding	180	Zinc
Metal binding	185	Calcium 3
Metal binding	186	Calcium 3; via carbonyl oxygen
Metal binding	193	Zinc
Metal binding	200	Calcium 2; via carbonyl oxygen
Metal binding	202	Calcium 2; via carbonyl oxygen
Metal binding	204	Calcium 2
Metal binding	206	Zinc
Metal binding	208	Calcium 3
Metal binding	209	Calcium
Metal binding	211	Calcium 3
Metal binding	403	Zinc 2; catalytic
Metal binding	407	Zinc 2; catalytic
Metal binding	413	Zinc 2; catalytic
Metal binding	476	Calcium 4; via carbonyl oxygen
Metal binding	521	Calcium 4; via carbonyl oxygen
Metal binding	569	Calcium 4; via carbonyl oxygen
Metal binding	618	Calcium 4; via carbonyl oxygen

## References

[1] Sambrook PN, MacGregor AJ, Spector TD (1999). Genetic influences on cervical and lumbar disc degeneration: a magnetic resonance imaging study in twins. Arthritis Rheum.

[2] Sobajima S, Kim JS, Gilbertson LG, Kang JD (2004). Gene therapy for degenerative disc disease. Gene Ther.

[3] Wallach CJ, Kim JS, Sobajima S (2006). Safety assessment of intradiscal gene transfer: a pilot study. Spine J.

[4] Beltran S, Angulo M, Pignatelli M, Serras F, Corominas M (2007). Functional dissection of the ash2 and ash1 transcriptomes provides insights into the transcriptional basis of wing phenotypes and reveals conserved protein interactions. Genome Biol.

[5] Sohn P, Cox M, Chen D, Serra R (2010). Molecular profiling of the developing mouse axial skeleton: a role for Tgfbr2 in the development of the intervertebral disc. BMC Dev Biol.

[6] Pache RA, Zanzoni A, Naval J, Mas JM, Aloy P (2008). Towards a molecular characterisation of pathological pathways. FEBS Lett.

[7] Thorn CF, Whirl-Carrillo M, Klein TE, Altman RB (2007). Pathway-based approaches to pharmacogenomics. Current Pharmacogenomics.

[8] Cloyd JM, Elliott DM (2007). Elastin content correlates with human disc degeneration in the anulus fibrosus and nucleus pulposus. Spine (Phila Pa 1976).

[9] Yu J, Fairbank JC, Roberts S, Urban JP (2005). The elastic fiber network of the anulus fibrosus of the normal and scoliotic human intervertebral disc. Spine (Phila Pa 1976).

[10] Matsui Y, Wu JJ, Weis MA, Pietka T, Eyre DR (2003). Matrix deposition of tryptophan-containing allelic variants of type IX collagen in developing human cartilage. Matrix Biol.

[11] Lamb J, Crawford ED, Peck D (2006). The Connectivity Map: using gene-expression signatures to connect small molecules, genes, and disease. Science.

[12] Yeh CT, Wu AT, Chang PM Trifluoperazine, an antipsychotic agent, inhibits cancer stem cell growth and overcomes drug resistance of lung cancer. Am J Respir Crit Care Med.

[13] Zhou Y, Jiang S, Chen J, Wang T, Jiang D, Chen H, Yu H (2013). 32k Da protein improve ovariectomy-induced bone loss in rats. Prion.

[14] Troyanskaya O, Cantor M, Sherlock G (2001). Missing value estimation methods for DNA microarrays. Bioinformatics.

[15] Fujita A, Sato JR, de Rodrigues LO, Ferreira CE, Sogayar MC (2006). Evaluating different methods of microarray data normalization. BMC Bioinformatics.

[16] Smyth GK, Michaud J, Scott HS (2005). Use of within-array replicate spots for assessing differential expression in microarray experiments. Bioinformatics.

[17] Zhang B, Kirov S, Snoddy J (2005). WebGestalt: an integrated system for exploring gene sets in various biological contexts. Nucleic Acids Res.

[18] Duncan DT, Prodduturi N, Zhang B (2010). WebGestalt2: an updated and expanded version of the Web-based Gene Set Analysis Toolkit. BMC Bioinformatics.

[19] Patil A, Nakamura H (2005). Filtering high-throughput protein-protein interaction data using a combination of genomic features. BMC Bioinformatics.

[20] Breitkreutz BJ, Stark C, Tyers M (2003). Osprey: a network visualization system. Genome Biol.

[21] Willis RC, Hogue CW (2006). Searching, viewing, and visualizing data in the Biomolecular Interaction Network Database (BIND). Curr Protoc Bioinformatics.

[22] Breitkreutz BJ, Stark C, Tyers M (2003). The GRID: The General Repository for Interaction Datasets. Genome Biol.

[23] Albert R, Jeong H, Barabasi AL (2000). Error and attack tolerance of complex networks. Nature.

[24] Huang da W, Sherman BT, Lempicki RA (2009). Bioinformatics enrichment tools: paths toward the comprehensive functional analysis of large gene lists. Nucleic Acids Res.

[25] Huang da W, Sherman BT, Lempicki RA (2009). Systematic and integrative analysis of large gene lists using DAVID bioinformatics resources. Nat Protoc.

[26] Wen Z, Wang Z, Wang S (2011). Discovery of molecular mechanisms of traditional Chinese medicinal formula Si-Wu-Tang using gene expression microarray and connectivity map. PLoS One.

[27] Leland WE, Taqqu MS, Willinger W, Wilson DV (1994). On the self-similar nature of Ethernet traffic. IEEE/ACM Transactions on Networking (TON).

[28] Papagiannaki K, Taft N, Zhang ZL, Diot C (2005). Long-term forecasting of internet backbone traffic. IEEE Trans Neural Netw.

[29] Suzek BE, Huang H, McGarvey P, Mazumder R, Wu CH (2007). UniRef: comprehensive and non-redundant UniProt reference clusters. Bioinformatics.

[30] Ding F, Shao ZW, Xiong LM (2013). Cell death in intervertebral disc degeneration. Apoptosis.

[31] Vadalà G, Russo F, Di Martino A, Denaro V (2013). Intervertebral disc regeneration: from the degenerative cascade to molecular therapy and tissue engineering. J Tissue Eng Regen Med.

[32] Haro H, Crawford HC, Fingleton B (2000). Matrix metalloproteinase-3-dependent generation of a macrophage chemoattractant in a model of herniated disc resorption. J Clin Invest.

[33] Roberts S, Caterson B, Menage J, Evans EH, Jaffray DC, Eisenstein SM (2000). Matrix metalloproteinases and aggrecanase: their role in disorders of the human intervertebral disc. Spine (Phila Pa 1976).

[34] Meijenhorst GC, de Bruin JN (1980). Hexabrix (ioxaglate), a new low osmolality contrast agent for lumbar epidural double-catheter venography. Neuroradiology.

[35] Grang L, Gaudin P, Trocme C, Phelip X, Morel F, Juvin R (2001). Intervertebral disk degeneration and herniation: the role of metalloproteinases and cytokines. Joint Bone Spine.

[36] Hristova GI, Jarzem P, Ouellet JA (2011). Calcification in human intervertebral disc degeneration and scoliosis. J Orthop Res.

